# Current advances in alteration of fatty acid profile in *Rhodotorula toruloides*: a mini-review

**DOI:** 10.1007/s11274-023-03595-3

**Published:** 2023-06-26

**Authors:** Chih-Chan Wu, Kohsuke Honda, Fujiyama Kazuhito

**Affiliations:** 1 International Center for Biotechnology, Osaka University, 2-1 Yamada-Oka, Suita, Osaka 565-0871 Japan; 2 Industrial Biotechnology Initiative Division, Institute for Open and Transdisciplinary Research Initiatives, Osaka University, 2-1 Yamadaoka, Suita, Osaka 565-0871 Japan

**Keywords:** Microbial lipid, Fatty acid alteration, Metabolic engineering, *Rhodotorula toruloides*

## Abstract

Microbial lipids are considered promising and environmentally friendly substitutes for fossil fuels and plant-derived oils. They alleviate the depletion of limited petroleum storage and the decrement of arable lands resulting from the greenhouse effect. Microbial lipids derived from oleaginous yeasts provide fatty acid profiles similar to plant-derived oils, which are considered as sustainable and alternative feedstocks for use in the biofuel, cosmetics, and food industries. *Rhodotorula toruloides* is an intriguing oleaginous yeast strain that can accumulate more than 70% of its dry biomass as lipid content. It can utilize a wide range of substrates, including low-cost sugars and industrial waste. It is also robust against various industrial inhibitors. However, precise control of the fatty acid profile of the lipids produced by *R. toruloides* is essential for broadening its biotechnological applications. This mini-review describes recent progress in identifying fatty synthesis pathways and consolidated strategies used for specific fatty acid-rich lipid production via metabolic engineering, strain domestication. In addition, this mini-review summarized the effects of culture conditions on fatty acid profiles in *R. toruloides*. The perspectives and constraints of harnessing *R. toruloides* for tailored lipid production are also discussed in this mini-review.

**Graphical abstract:**

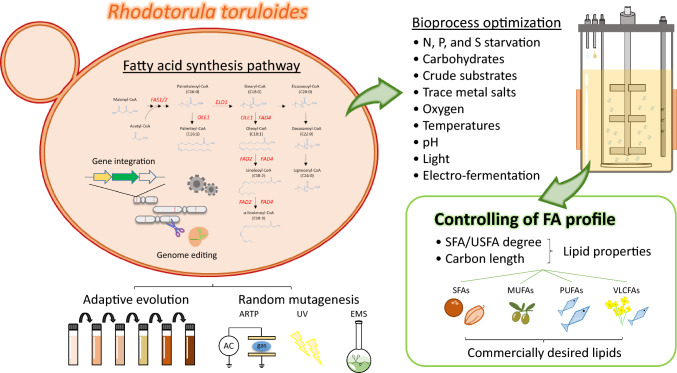

## Introduction

Owing to the depletion of petroleum storage and the growing world population, lipids derived from plants and animals have been exploited as an alternative to petroleum for producing biofuel and oleochemicals (Chemat et al. 2021; Ndiaye et al. 2019; Riazi et al. 2020; Shaah et al. 2021). However, production limitations (long harvesting time and unstable production affected by geographical and climate factors) and environmental issues (environmental pollution and competence of arable land for food crops) of plant- and animal-based lipids have forced scientists to seek new alternatives (Singh et al. 2020a; Meijaard et al. 2020; Toldrá-Reig et al. 2020). To date, much attention has been paid to the development of microbial lipids because of their short harvesting time and fewer environmental concerns (Bao et al. 2021; Liu et al. 2021c; Aamer Mehmood et al. 2021; Uthandi et al. 2021).

*Rhodotorula toruloides* is a non-conventional red yeast, also known as *Rhodosporidium toruloides*, *R. rubescens*, *R. glutinis*, or *R. gracilis*, that belongs to the subphylum *Pucciniomycotina* within the division *Basidiomycota* (Abeln and Chuck 2021; Koh et al. 2014). This strain is an excellent microbial lipid producer and has received great attention owing to its outstanding lipogenic ability. *R. toruloides* can accumulate up to 70% of its dry cellular weight as lipid content under nutrient-limiting conditions (nitrogen, phosphate, and sulfate) (Wang et al. 2018; Wu et al. 2010, 2011). It is also well known for its robustness, as it can produce lipids by utilizing a wide range of substrates from low-cost industrial by-products (Zhao et al. 2022; Wen et al. 2020; Saini et al. 2020; Park et al. 2018b). Numerous studies have described ways to improve lipid production in *R. toruloides*, including robust mutant selection, bioprocess optimization, and metabolic engineering (Zhao et al. 2022; Wen et al. 2020; Saini et al. 2020; Park et al. 2018b). In addition, available genome sequence (Dinh et al. 2019; Martín-Hernández et al. 2021; Zhu et al. 2012), characterized lipid metabolism (Jagtap et al. 2021), and accessible genetic tools (Bonturi et al. 2022; Jiao et al. 2019; Otoupal et al. 2019) for *R. toruloides* have also been established and developed. Two efficient and time-saving transformation methods by using Lithium-acetate (Tsai et al. 2017) and electroporation (Liu et al. 2017) were established to shorten the experimental time of the traditional method via *Agrobacterium tumefaciens*-mediated transformation (ATMT) (Lin et al. 2014). In addition, RNAi machinery was also proven to be functional for gene downregulation in *R. toruloides* (Liu et al. 2019). Genome editing toolboxes such as CRISPR-Cas9 system were also reported to achieve single or multi-genes editing in *R. toruloides* (Jiao et al. 2019; Otoupal et al. 2019; Schultz et al. 2019). All these advanced genetic tools and knowledge accelerated the improvement of the control of lipid production in *R. toruloides* and broaden its applications.

Microbial lipids rich in triacylglycerols are promising and attractive alternatives to existing petroleum-, plant-, and animal-based lipids. A triacylglycerol molecule consists of three fatty acid (acyl) chains attached to an alcohol glycerol backbone (Yoshinaga 2021). Carbon length, desaturation level, and position determine the physicochemical and biological properties of fatty acid (FA) chains attached to triacylglycerols (Cook and McMaster 2002; Temkov and Muresan 2021; Lee et al. 2022; Saini et al. 2021; Falomir-Lockhart et al. 2019). The lipids from *R. toruloides* have FA compositions that are similar to those of plant-derived oils. Despite the variation of fatty acid profiles existing in different *R. toruloides* strains, lipids produced in *R. toruloides* mainly consist of C16-C18 long-chain FAs (palmitic acid [PA], C16:0, 15–40% of total fatty acid (TFA); palmitoleic acid [POA], C16:1, 1–2% of TFA; stearic acid [SA], C18:0, 10–20% of TFA; oleic acid [OA], C18:1, 40–60% of TFA; linoleic acid [LA], C18:2, 1–10% of TFA; α-linolenic acid [ALA], C18:3, 1–2% of TFA). In addition, a small proportion of FAs, such as myristic acid (MA, C14:0, 2% of TFA), arachidic acid (AA, C20:0, less than 0.5% of TFA), docosanoic acid (DA, C22:0, less than 0.5% of TFA), and tetracosanoic acid (TA, C24:0, less than 1% of TFA), have also been reported in *R. toruloides* (Krikigianni et al. 2022; Zhang et al. 2022a; Liu et al. 2021a). Therefore, *R. toruloides*-derived lipids could potentially be exploited as feedstock for plant oil substitutes in different applications, such as biodiesel, biolubricants, cosmetics, nutritional supplements, plastics, and coating materials (Carmona-Cabello et al. 2021; Papadaki et al. 2018; Adrio 2017; Lopez-Huertas 2010; Yu et al. 2014). For example, lipids rich in OA (over 70% of TFA) are preferred for biodiesel production due to their appropriate fluidity and stability during storage (Graef et al. 2009). Lipids with high saturated FA profiles are desired as equivalents to coconut (PA, 23–30%; SA, 32–37%; OA, 30–37%; LA, 2–4% of TFA) and cocoa (PA, 26%; SA, 35%; OA, 33%; LA, 3% of TFA) butter in food industries (Lipp et al. 2001; Papanikolaou and Aggelis 2010). Moreover, lipids rich in polyunsaturated fatty acids (PUFA) such as docosahexaenoic acid (C22:6, DHA) and eicosapentaenoic acid (C20:5, EPA) (16% and 17% of TFA) are considered as substitutes to fish oils (Lee et al. 2019). Lipids containing conjugated linoleic acids (CLAs, a group of isomers of LA), γ-linolenic acid (GLA, C18:3), and nervonic acid (NA, C24:1), which are relatively low in their sources, are considered as valuable lipids because of their clinical benefits (Szczepańska et al. 2022). However, commercially desired lipids are often produced at low concentrations or with inappropriate FA compositions in microbial lipids, which may lead to complex downstream processes and result in high production costs (Ochsenreither et al. 2016; Barta et al. 2021). Therefore, harnessing *R. toruloudes* for specific FA-rich lipid production is a vital issue for industrialization. Most studies have focused on maximizing the lipid-producing ability of *R. toruloides* (Zhao et al. 2022; Wen et al. 2020). Hence, few review papers have summarized and discussed strategies for controlling FA composition in *R. toruloides*. Further, a thorough discussion of the culture conditions that affect FA profiles in *R. toruloides* has not yet been done. In this mini-review, we summarize the advances in the control and production of tailored lipids and the effects of culture conditions on FA profiles in *R. toruloides* (Table 1).

**Table 1:** Approaches used to alter FA composition in *R. toruloides*

Approach	Strategy	Parental strain	Carbon source	Major FA profile	References
*Metabolic* *engineering*	Overexpression of *RtELO1*	CECT13085	Glycerol	Increased OA from 50% to 60–70% of TFA	Fillet et al. (2016)
	Overexpression of *RtELO1*	ATCC 10657	Glucose	Increased OA from 50% to 60–70% of TFA	Liu et al. (2018)
	Gene disruption of *RtELO2*	ATCC 10657	Glucose	No change in FA profile	Liu et al. (2018)
	Overexpression of genomic *RtOLE1*	TK16-DMKU3	Glucose	Increased OA from 50 to 62% of TFA	Tsai et al. (2019)
	Gene disruption of *RtFAD2*	ATCC 10657 (Δku70e)	Glucose	Increased OA from 30 to 60% of TFA	Liu et al. (2021b)
	Overexpression of genomic *RtFAD2*	TK16-DMKU3	Glucose	Increased LA from 14 to 28% and ALA from 1.2% to 3.9% of TFA	Wu et al. (2021)
	Co-overexpression of codon-optimized *MaFAD2* and *FvFAD2*	AS 2.1389	Glucose	Increased LA from 5 to 27% of TFA	Wang et al. (2016)
	Disruption of *RtALD1* and overexpression of codon-optimized *MaFAD2*	ATCC 10657	Glucose	Increased LA	Liu et al. (2018)
	Disruption of *RtFAD2* and co-overexpression of codon-optimized *MaFAD2* and *MaFAD6*	ATCC 10657 (Δku70e)	Glucose	Increased OA to 60.1% of TFA and produced 27.3% GLA of TFA	Liu et al. (2021b)
	Co-overexpression of plant derived *KCS*s and *OLE1*	CECT 13085	Glucose	Produced EA and NA (20% of TFA)	Fillet et al. (2017)
*Mutant* *isolation*	ALE at 37°C	TK16-DMKU3	Glucose	Mutant L1-1 produced 86% OA of TFA at 37°C	Wu et al. (2018)
	ALE with wheat straw hydrolysate	NRRL Y-1091	Glucose, xylose	Mutant CH4 and CH5 produced 40–47% OA and 3.5% LA of TFA	Liu et al. (2021d)
	UV mutagenesis and selected mutant under ethanol and H_2_O_2_ or cerulenin	NBRC 8766	Glucose	Increased PA (5.6–6.5%) and SA (12.7–13.5%) of TFA	Yamada et al. (2017)
	UV mutagenesis and selected mutant under ethanol and H_2_O_2_ or LiCl	2.1389	Glucose	Increased PA (23–24%) and SA (10–12%) of TFA	Guo et al. (2019)
	ARTP and NTG mutagenesis and selected mutant by color	NP11	Glucose	No change in FA profile	Zhang et al. (2016a)
	ARTP followed by ALE with tea waste hydrolysate	ACCC 20341	Tea waste hydrolysate	Increased 5.5% ALA of TFA	Qi et al. (2020)
*Optimization of* *bioprocess*	Optimized C/N ratio	R-ZL mutant (from AS 2.1389)	Sucrose	Increased PA (21–26% of TFA) and ALA (6–15% of TFA)	Ye et al. (2021)
	Optimized C/P ratio	Y4 mutant (from AS 2.1389)	Glucose	Increased OA from 50 to 67% of TFA	Wu et al. (2010)
	Optimized C/S ratio	Y4 mutant (from AS 2.1389)	Glucose	Increased saturated FA (MA, PA and SA) up to 63% of TFA	Wu et al. (2011)
	Used C5 sugars (arabinose and xylose)	CBS14 (from CBS)	Arabinose or/and xylose	Slightly increased PA (25–30% of TFA)	Wiebe et al. (2012)
	Used crude glycerol as a carbon source	ATCC 10788	Crude glycerol	Increased 40% saturated FAs (PA and SA) of TFA	Uperty et al. (2017, 2018)
	Used acetic acid as a carbon source and optimized C/N ratio	AS 2.13	Acetic acid	Slightly increased OA to 50% of TFA from 42% of TFA while using glucose	Huang et al. (2016)
	Used propionic acid (C3) as a carbon source	NCYC 1576	Propionic acid	Produced 31% of odd-chain fatty acids (C17:0 and C17:1) of TFA	Krikigianni et al. (2022)
	Applied essential oils from plants	ATCC 10788	Glycerol and limonene	EOs from clove, cinnamon pine, and orange increase SA or PA contents	Uprety and Rakshit (2018)
	Supplemented with Mg^2+^	1588	Wood hydrolysate	Produced ETAs (0.03% of TFA)	Saini et al. (2022a)
	Supplemented with Cu^2+^	1588	Wood hydrolysate	Produced DHA (0.05% of TFA) and GLA (0.12% of TFA)	Saini et al. (2022a)
	Supplemented with Zn^2+^or Fe^2+^	1588	Wood hydrolysate	Produced GLA (0.2% of TFA)	Saini et al. (2022a)
	Optimized oxygen availability	NCYC 921	Carob pulp syrup	Content of PUFAs elevated as oxygen availability increased	Parreira et al. (2015)
	Increased cultivation temperature to 37°C	TK16-DMKU3	Glucose	Increased the content of saturated FAs (PA and SA) content (30% from 17%)	Wu et al. (2020)
	Decreased cultivation temperature to 15°C	YM25079	Glucose	The content of LA and ALA increased from 22 and 8% to 35% and 21% of TFA	He et al. (2015)
	Assessed the pH effects on FA profile	NCYC 921	Glucose	FA profiles slightly changed at various pH	Dias et al. (2016)
	Exposed light during cultivation	NBRC 10032	Glucose	Increased POA and ALA to 3.6% and 4.5% of TFA from 1.6% and 1.1% of TFA	Pham et al. (2020)
	Electro-fermentation with redox mediator Neutral Red	DSM 4444	Glucose	Increased the content of saturated FAs (from 37 to 50% of TFA)	Arbter et al. (2019)

*PA* palmitic acid, *POA* palmitoleic acid, *SA* stearic acid, *OA* oleic acid, *LA* linoleic acid, *ALA* α-linolenic acid, *GLA* γ-linolenic acid, *DHA* docosahexaenoic acid, *EA* erucic acid, *ETA* eicosatrienoic acid, *NA* nervonic acid, *PUFA* polyunsaturated fatty acid, *TFA* total fatty acid

## Harnessing de novo FA synthesis pathways in *R. toruloides*

**Fig. 1:**
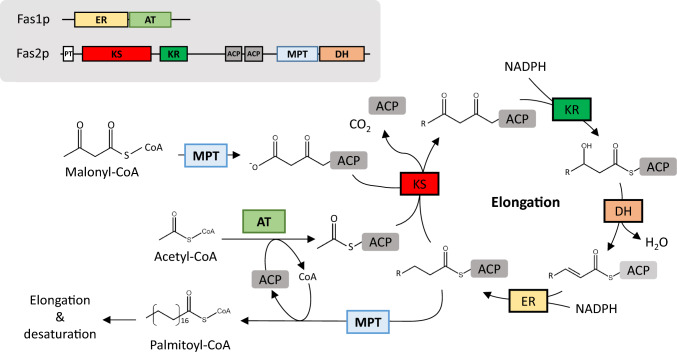
Schematic diagram of fatty acid synthase and fatty acid synthesis in *R. toruloides* cells. Abbreviations indicate components of the fatty acid synthase system. *ACP* acyl carrier protein, *AT* acyltransferase, *DH* dehydratase, *ER* enoyl reductase, *KR* ketoacyl reductase, *KS* ketoacyl synthase, *MPT* malonyl/palmitoyl transferase, *PT* phosphopantetheine transferase

The FA composition of yeasts is precisely synthesized and regulated by FA synthases (Fass), elongases (Elos), and desaturases (Fads) (Heil et al. 2019; Singh et al. 2020b; Takaku et al. 2020; Matsuzawa et al. 2020). FA synthase, an essential enzyme in FA synthesis, catalyzes the reaction between acetyl-CoA and malonyl-CoA to produce C16-C18 FAs (Heil et al. 2019). *R. toruloides* Fas (*Rt*Fas) consists of two subunits (α- and β-subunits), which are encoded by *FAS1* (*RtFAS1*) and *FAS2* (*RtFAS2*), to form a multifunctional enzyme (Fischer et al. 2015; Zhu et al. 2012) (Fig. 1). *Rt*Fas1 contains acyltransferase and enoyl reductase domains. *Rt*Fas2 contains phosphopantetheinyl transferase, ketoacyl synthase, ketoacyl reductase, two acyl carrier proteins, malonyl/palmitoyl transferase, and dehydratase domains (Fig. 1). Liu et al. (2019) demonstrated that knocking down *RtFAS1* and *RtFAS2* using RNA interference decreased lipid content, but did not change the FA profile in *R. toruloides* NP11. Heterologous expression of *Rt*Fas in *Saccharomyces cerevisiae* also showed no effect on the FA composition (Zhou et al. 2016). These studies imply that *FAS* may not be a good candidate for manipulating FA composition in *R. toruloides*. However, enzyme engineering of the KS domain of Fas in the oleaginous yeast *Yarrowia lipolytica* enhanced the content of medium chain FAs (Rigouin et al. 2017), which suggests that engineering Fas may be applied in *R. toruloides* to modify the FA composition*.*

**Fig. 2:**
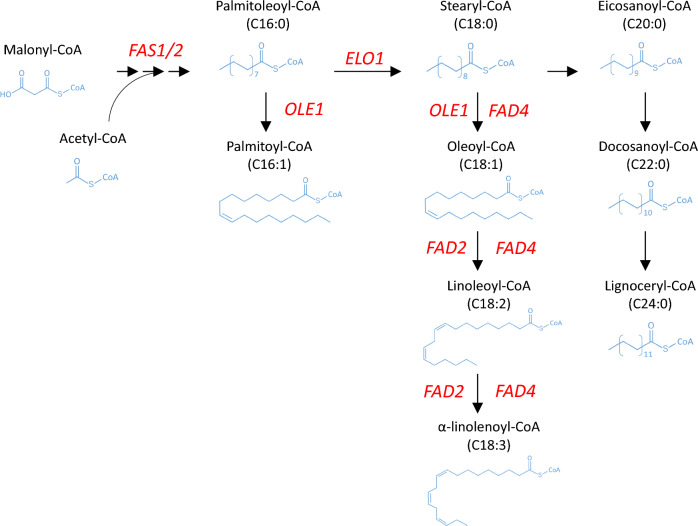
Fatty acid elongation and desaturation pathways in *R. toruloides*. Genes encoding FA synthase, elongases, and desaturases are in red

Once malonyl-CoA is elongated to the carbon length of the acyl chain to C16, C16 acyl-CoA is released from the barrel-shaped Fas in *R. toruloides*. The released C16 acyl-CoA is further modified by FA Elos and Fads in *R. toruloides* (Zhu et al. 2012) (Fig. 2). Fads catalyze the desaturation of FAs by introducing double bonds into C–C bonds, which generate monounsaturated FAs (MUFAs) and polyunsaturated FAs (PUFAs) (Takaku et al. 2020; Wang et al. 2022a). Elos mediate the elongation of FAs by adding extra carbon atoms to the acyl chains. All desaturases and elongases exhibit different substrate specificities and spatial and temporal characteristics (Cerone and Smith 2022; Szczepańska et al. 2022). Therefore, elucidation of the functions of Fads and Elos could assist in the control of FA composition and production of customized lipids in *R. toruloides*.

The functions of Elos have been well studied in the model yeast *S. cerevisiae* and other yeasts used in the industry (Kihara 2012; Uemura 2012; Rigouin et al. 2018; Matsuzawa et al. 2020); however, few studies have elucidated the functions of Elos in *R. toruloides*. A patent (Fillet et al. 2016) reported that overexpression of *RtELO1* significantly enhanced the OA content to 70% of the TFA in *R. toruloides*. In another patent (Liu et al. 2018), overexpression of *RtELO1* led to an increase in OA. In addition, disruption of *RtELO2* did not affect FA profiles (Liu et al. 2018). Conversely, studies on *Rt*Fads have also been conducted. *R. toruloides* Δ9-desaturase (*Rt*Ole1) catalyzes the conversion of SA to OA by introducing a *cis*-double bond at the Δ9 position of acyl-CoA. OA, one of the most essential and abundant FA in *R. toruloides*, provides appropriate fluidity and excellent thermal and oxidative stability for oleochemical applications such as biodiesel, biolubricants, and hydraulic fluids (Wang et al. 2022b). OA production is determined by *Rt*Ole1, which is a rate-limiting enzyme in LA and ALA biosynthesis (Nagao et al. 2019). Hence, *Rt*Ole1 plays a vital role in controlling the FA profiles. Overexpression of *Rt*Ole1 in *R. toruloides* have been shown to effectively increase OA content up to 70% and only slightly increase POA content (Tsai et al. 2019). Liu et al. (2021b) suggested that *Rt*Ole1 has a strong substrate preference for stearoyl-CoA over palmitoyl-CoA. In addition, blocking the downstream desaturation of OA into LA by disrupting *RtFAD2* could also increase the OA content from 30 to 60% TFA. LA and ALA are essential FAs that humans cannot synthesize. Hence, lipids with high LA and ALA contents have high value as nutraceuticals in the human diet (Chen and Liu 2020). Liu et al. (2021b) also demonstrated that *R. toruloides FAD2* and *FAD4* encode Δ12/Δ15 desaturase and ∆9/Δ12/Δ15 desaturase, respectively, which produce LA and ALA from OA by introducing C–C double bonds at the Δ12 and Δ15 positions of FAs. Additionally, the co-overexpression of *RtOLE1* and *RtFAD2* has been applied to produce LA-rich lipids (Wu et al. 2021). Nevertheless, downregulation of *RtOLE1* expression by elevated LA and ALA was observed in *R. toruloides* (Wu et al. 2021). Furthermore, two regulatory elements, ORE1 and ORE2, upstream of *RtOLE1* have been reported (Liu et al. 2021b). The authors suggested that ORE1 positively regulates *R. toruloides FAD* gene transcription. Therefore, elucidating the transcription factors associated with the regulatory elements of *RtOLE1* is necessary to manipulate the FA desaturation pathway. For example, *S. cerevisiae* transcriptional factors Spt2 and Mga2 upregulate essential genes involved in unsaturated FA biosynthesis (Zhang et al. 2022b; Sinha et al. 2022). The oleaginous *Y. lipolytica* Mga2 protein also regulates desaturase gene expression (Liu et al. 2015). Taken together, these studies show that manipulating the endogenous FA synthesis pathway in *R. toruloides* is an effective strategy for tailoring lipid production. Furthermore, the regulation of the native FA synthesis pathway should also be considered for a rational design to enhance specific FA production.

## Metabolic engineering by introduction of exogenous genes for specific high-value lipid production

In addition to manipulating the native FA synthesis pathway, overexpression of genes from plants or other fungi was attempted in *R. toruloides* to produce specific high-value lipids. Disrupting native aldehyde dehydrogenase (*ALD1*) and overexpressing *FAD2* from *Mortierella alpina* and *FAD3* from *Aleurites fordiiek* led to a total ALA content of up to 49% of TFA (Liu et al. 2018). Tsai et al. (2019) enhanced the OA content from 50 to 70% of TFA by introducing *S. cerevisiae OLE*, which encodes a Δ9-desaturase. Wang et al. (2016) successfully enhanced LA content five-fold and achieved final LA titers of up to 1.3 g/L under flask culture conditions by introducing *FAD2* from *M. alpina* and *Fusarium verticillioides* (Wang et al. 2016). GLA is a valuable omega-6 FA predominantly present in plants in relatively low amounts. GLA has anti-inflammatory properties and has applications in the treatment of several diseases (Liu et al. 2021b). Furthermore, Liu et al. (2021b) succeeded in producing GLA by disrupting native *RtFAD2* and co-overexpressing *M. alpine FAD2* and *FAD6*. The OA and GLA contents were further increased to 60.1% and 27.3% of TFA, respectively, in 2-L bioreactors.

FAs with carbon lengths of 20 or more are known as very-long-chain FAs (VLCFAs). VLCFAs, such as erucic acid (EA, C22:1) and NA, are potentially renewable feedstocks for the production of plastics, cosmetics, resins, nylon, and lubricants (Fillet et al. 2017). Additionally, NA has the potential to be used in the treatment of several neurological diseases (Liu et al. 2021a). Hence, EA and NA are high-value FAs. However, *R. toruloides* cannot naturally produce either EA or NA. To produce VLCFA-rich lipids, Fillet et al. (2017) introduced various plant-derived 3-ketoacyl-CoA synthases (*KCS*s) into *R. toruloides* and successfully produced EA and NA. By increasing the *KCS* copy number and co-overexpressing plant-derived *ELO*s, the engineered strain produced more EA and NA, which accounted for 27% of TFA in 7-L bioreactors.

## Domestication of *R. toruloides* strains for tailored lipid production

Although metabolic engineering is a powerful strategy for harnessing *R. toruloides* for tailored lipid production, lack of information of the genetic background is the main limitation for strain engineering. Therefore, other strategies, such as adaptive laboratory evolution (ALE) and mutagenesis by physical/chemical methods, are also popular for isolating mutants with the desired traits for tailored lipid production in *R. toruloides* (Wen et al. 2020). The mutation rate is one of the main differences between ALE and mutagenesis using physicochemical methods. ALE domesticates parental strains with simultaneous mutation rates by growing several generations under selective pressure conditions (Phaneuf et al. 2020). Mutagenesis by physical and chemical methods commonly introduces a high mutation rate by random DNA double-strand breakage with UV irradiation and chemical agents. Although mutagenesis by physical and chemical methods is an effective way to introduce mutations into the genome, isolated mutants usually possess undesired defects (Arora et al. 2020). Owing to the advantages and disadvantages of the two methods, researchers have employed ALE and mutagenesis by physical and chemical methods in *R. toruloides*.

Wu et al. (2018) attempted to overcome the high temperatures that occur during fermentation by isolating thermotolerant *R. toruloides* DMKU3-TK16. The thermotolerant strain L1-1 was isolated under heat stress at 37°C using the ALE method. The isolated strain exhibited improved growth and lipid productivity at 37°C and a high OA content (90%) when cultured at 37°C. Liu et al. (2021d) attempted to improve the tolerance of *R. toruloides* to toxic lignocellulosic hydrolysates for lipid production. The isolated strains showed improved growth in media containing lignocellulosic biomass hydrolysate. According to their data, the evolved strains CH4 and CH5 showed improved OA and LA content (calculated). However, most evolved strains did not show significant FA profile alterations compared to the parental strain (*R. toruloides* NRRL Y-1091). Mutagenesis by UV has also been used for strain improvement in *R. toruloides*. Yamada et al. (2017) attempted to improve the lipid-producing ability of *R. toruloides* NBRC 8766 by UV irradiation and mutant selection under ethanol and H_2_O_2_ or cerulenin stress. Two strains with improved lipid production (8766 2-31 M and 8766 3-11C) were isolated. The FA profiles of these two strains showed considerably increased PA content (12.7% and 13.5%) with decreased MA and LA contents. Another study adopted a similar strategy of UV mutagenesis followed by mutant selection under ethanol and H_2_O_2_ or LiCl stress (Guo et al. 2019). The research team obtained two strains (R-ZL2 and R-ZL13) with improved lipid production from *R. toruloides* strain 2.1389. The R-ZL2 and R-ZL13 strains exhibited higher saturated FA profiles with increased PA (23 and 24%) and SA (12 and 10%) content, respectively. These two studies demonstrated that UV mutagenesis combined with stress selection could effectively domesticate *R. toruloides* to produce FA-rich lipids. Zhang et al. (2016a) obtained mutants of *R. toruloides* NP11 using atmospheric and room temperature plasma (ARTP) and nitrosoguanidine (NTG) methods. However, they did not apply stress selection after mutagenesis. According to their results, the isolated mutant XR-2 did not show a significant change in the FA profile compared with the parental strain *R. toruloides* NP11. In contrast, Qi et al. (2020) applied stress selection using inhibitory lignocellulosic hydrolysates after ARTP. Mutant RM18 exhibited improved ALA content (5.5%, 1.6 times more than the parental strain). These studies (Yamada et al. 2017; Guo et al. 2019; Zhang et al. 2016a; Qi et al. 2020) imply that stress selection after physical or chemical mutagenesis is necessary for screening mutants for desired FA production.

## Culture conditions affect FA profiles in *R. toruloides*

Numerous strategies, such as optimizing culture conditions and waste utilization, have been applied to enhance lipid production in *R. toruloides* to produce economically competitive lipids (Zhao et al. 2022). However, the effects of each strategy on the FA profiles of lipids varied. Therefore, we have consolidated the progress obtained and provided a wide scope in the following sections. The information can help design rational bioprocesses to enhance specific FA-rich lipid production and assist in tailored lipid production.

### Nitrogen, phosphate, and sulfate starvation

Nitrogen (N), phosphate (P), and sulfate (S) starvation can promote lipid accumulation in *R. toruloides* (Wang et al. 2018; Wu et al. 2010, 2011), and nitrogen limitation is the most adapted method. Different strategies affect lipid synthesis via different pathways, resulting in different metabolic fluxes. Therefore, the N, P, and S concentrations also affected the FA profiles. Ye et al. (2021) found that, when using sucrose as the sole carbon source and adjusting the C/N ratio to over 80 with ammonium sulfate or ammonium nitrate in the culture media, the FA contents of PA and ALA significantly increased in cells. Increasing the C/P ratio with glucose as a carbon source led to an increase in the OA content from 50 to 67% (Wu et al. 2010). In a study of S-limitation (Wu et al. 2011), lipids produced under increased C/S conditions favored saturated FAs (MA, PA, and SA) up to 60%. According to these studies, the strategies used to trigger lipid production should focus on enhancing the specific FA content.

### Carbohydrates

Carbohydrates significantly affect FA profiles by activating key metabolic genes involved in gluconeogenesis, the glyoxylate cycle, and the tricarboxylic acid cycle (Sun et al. 2021; Patel et al. 2015). Most microbes possess a unique carbon catabolite mechanism. Microbes metabolize sugars sequentially because glucose represses the utilization of other sugars (Sun et al. 2021; Patel et al. 2015). Therefore, strategies using a primary carbon substrate or a defined ratio of carbon substrates have been reported for *R. toruloides*. Wiebe et al. (2012) demonstrated that *R. toruloides* could utilize C5 (arabinose and xylose) and C6 (glucose) sugars for lipid production. The lipids produced using arabinose and xylose as the sole carbon sources showed increased PA and LA contents compared to glucose, indicating that C5 (xylose and arabinose) sugars significantly changed the FA profiles. However, the FA profiles of the lipids produced using mixed sugars of glucose, arabinose, and xylose did not change significantly compared to those produced using glucose alone. Lignocellulosic biomass hydrolysate is a promising sustainable feedstock for microbial lipid production. After pretreatment and saccharification of lignocellulosic biomass, the complex links among lignin, cellulose, and hemicellulose are broken down, thus releasing fermentable sugars, such as glucose, xylose, or arabinose (Saini et al. 2022b). Osorio-González et al. (2019) demonstrated that *R. toruloides* strains had higher PA and SA contents when using C5 hydrolysate than C6 hydrolysate. Therefore, selecting carbohydrates and monitoring the carbohydrate content in the media during fermentation is vital for designing a rational fermentation process for desired FA production.

### Crude substrates

In addition to using a single carbon source or defined carbon source, non-monomeric substrates containing abundant carbon sources from industrial, agricultural, municipal solid, and biomass wastes have been extensively studied and employed for lipid production in *R. toruloides* owing to their low cost (Abeln and Chuck 2021; Zhao et al. 2022).

Crude glycerol (CG) is a widely available product in the biodiesel industry. Approximately 10% (w/w) of glycerol is generated in every batch of biodiesel and is considered a waste (Lee et al. 2014; Uprety et al. 2016). Uprety et al. (2017, 2018) indicated that *R. toruloides* cells grown in media containing CG contained nearly 40% saturated FA (PA and SA), and a reduction in OA (from 60 to 47% of TFA) was observed compared to cells grown with pure glycerol. CG usually consists of glycerol and other impurities, such as glycerol, soap, methanol, ash, FA methyl esters (FAME), and salt. Uprety et al. (2018) reported that the decrease in OA in cells resulted from various impurities, particularly soap. Therefore, impurities in CG should be noted for tailored lipid production.

*R. toruloides* can utilize volatile FAs (VFAs), such as acetic acid (C2), propionic acid (C3), and butyric acid (C4) (Gao et al. 2017) for lipid production. VFAs can be obtained through the degradation of organic waste biomass by anaerobic digestion (Park et al. 2018a; Llamas et al. 2020), and thus VFAs can be exploited as cheap carbon sources for lipid production in yeasts (Park et al. 2018a, b; Llamas et al. 2020). However, different VFAs caused different FA changes in *R. toruloides*. *R. toruloides* grown in media with acetic acid (C2) as the sole carbon source generates high OA-lipids (nearly 50% of TFA) (Huang et al. 2016; Krikigianni et al. 2022). When propionic acid (C3) was used as the sole carbon source, the lipids had a high content of saturated FAs (65.9% of TFA) and a low MUFA content of 16% (Krikigianni et al. 2022). Notably, when propionic acid C3 was used, odd-chain FAs (margaric acid, C17:0, and heptadecenoic acid, C17:1) were generated, accounting for 30.8% of TFA. When fermented brewer’s spent grain contained mixed VFAs, the production of odd-chain FAs (ODFAs) was not observed, indicating that VFA composition influenced the FA profiles in *R. toruloides*. ODFAs are unusual and rare FAs that are found in natural sources. They are valuable FAs because they are associated with several health benefits, such as regulating allergies, psoriasis, and autoimmune disorders, and reducing the risk of metabolic disorders (Dąbrowski and Konopka 2022). Therefore, using VFAs as a carbon source for lipid production in *R. toruloides* could alter the native FA profile and produce unusual FA.

Plant-derived essential oils (EOs) can also alter the FA profiles of oleaginous microbes (Uprety and Rakshit 2018; Uprety et al. 2022). EOs are volatile and hydrophobic compounds mainly composed of terpenic hydrocarbons and oxygenated derivatives. EOs can impact the carbon flux toward a specific type of FAs inside microbes. Hence, EOs have been applied to alter the FA profile of *R. toruloides* (Uprety and Rakshit 2018). The EOs from clove-, cinnamon-, pine-, and orange-supplemented growth media for *R. toruloides* significantly increased the saturated FA content to 29%, 15%, 14%, and 36% of TFA, respectively. Furthermore, the EOs from orange also elevated PA content to 41% of TFA (Uprety and Rakshit 2018). Therefore, EOs are good inducers of FA production in *R. toruloides*.

### Trace metal salts

Trace metal salts, such as zinc sulfate, copper sulfate, ferric chloride, manganese sulfate, and magnesium sulfate have also been commonly used as micronutrients in growth media to promote microbial growth (Saini et al. 2022a, b). These micronutrients serve as co-factors for several enzymes involved in lipid synthesis and desaturation pathways, and affect their catalytic activities (Singh et al. 2016; Ma et al. 2009; Romero et al. 2018). Furthermore, these metals may alter the catalytic activities of desaturase enzymes, resulting in alterations in the FA composition of lipids (Cai et al. 2020). Metal supplementation with wood hydrolysate as a carbon source causes the production of unusual PUFAs in *R. toruloides* (Saini et al. 2022a, b). When cells were grown in media supplemented with Mg^2+^, *R. toruloides* cells produced eicosatrienoic acid (ETA, C20:3) (0.03% of TFA). Similarly, an unusual DHA (0.05% of TFA) was produced by *R. toruloides* in media supplemented with Cu^2+^. In addition, adding Zn^2+^, Fe^2+^, and Cu^2+^ increased the content of GLA, which is also an unusual FA in *R. toruloides*, from 0.07% to 0.22%, 0.2%, and 0.12% of TFA, respectively. EA, DHA, and GLA are essential FA in humans; hence, they are high-value compounds. These results indicated that the addition of specific trace metals induced the production of high-value PUFAs in *R. toruloides*.

### Oxygen

Oxygen availability is a critical parameter, especially during high cell density and large-scale fermentation, because of the unequal oxygen distribution in the microenvironment of the vessels, which affects cell growth. Oxygen is also a key molecule involved in FA desaturation. Fads introduce C–C double bonds into acyl chains by reducing oxygen (Sperling et al. 2003). Because these are oxygen-dependent reactions, low oxygen availability results in a higher degree of lipid saturation (Abeln and Chuck 2021). In addition, oxygen levels affect the activation of crucial genes, such as *S. cerevisiae OLE1* in the FA saturation pathway through transcriptional factors (Romero et al. 2018; Burr et al. 2016). Therefore, oxygen is essential for the desaturation of FA by Fas. Choi et al. (1982) observed that the degree of unsaturation was significantly affected by the specific oxygen uptake rate of *R. toruloides*. Parreira et al. (2015) also confirmed that the PUFAs fraction increased as oxygen availability increased. These results indicated that oxygen availability is associated with PUFA synthesis in *R. toruloides*. Furthermore, the oxygen-activation pathways of Fas should be confirmed in *R. toruloides*.

### Temperature

The growth temperature greatly influences various life processes in organisms, including gene transcription, metabolic activity, cell growth, nutrient absorption, cell survival, energy production, and membrane fluidity (Price and Sowers 2004; Fonseca et al. 2019). Microbial cells can modulate membrane fluidity to adapt to environmental temperature. Temperature influences membrane fluidity; decreasing temperature results in reduced viscosity and a more rigid membrane, whereas increasing temperature leads to increased viscosity and a looser membrane (Fonseca et al. 2019; Renne and Kroon 2018). Hence, microbial cells change their FA composition in the membrane lipids to maintain membrane fluidity. An increase in unsaturated FAs content reduces the melting point and increases membrane fluidity (Chen et al. 2022). On the other hand, an increase in saturated FAs content elevates their melting point and decreases membrane fluidity (Mejía-Barajas et al. 2018).

A growth temperature of 30°C is typically adopted for lipid production in *R. toruloides*. However, higher and lower temperatures significantly influence the FA profile of *R. toruloides*. The FA profile of the lipids in *R. toruloides* cells grown at 37°C increased the content of saturated FAs (PA and SA) (30% from 17%), which led to a higher saturated level than that at 30°C (Wu et al. 2020). Notably, the increase in saturated FA at 37°C may result from reduced *Rt*Fad2 desaturase activity (Wu et al. 2021). These reports suggest that temperature affects Fad activity and alters membrane FA composition, which may help cells maintain membrane fluidity. In contrast, when *R. toruloides* cells were grown at 15°C, the content of PUFAs, including LA and ALA, increased significantly compared to that at 25°C. The LA and ALA contents increased from 22 to 35% and from 8 to 21%, respectively (He et al. 2015). He et al. (2015) also demonstrated that the mRNA transcription level of a putative FA desaturase was five-fold higher in cells grown at 15°C than in cells grown at 25°C. In *Y. lipolytica*, the transcriptional factor Mga2, encoded by YALI0B12342g, may be associated with the upregulation of *FAD2* at low temperatures (Tezaki et al. 2017). Therefore, elucidating the mechanism of low-temperature-induced *FAD2* expression is necessary for *R. toruloides* to produce PUFAs.

### pH

The commonly set pH for lipid production in *R. toruloides* is 5–6 (Li et al. 2006; Yang et al. 2014; Zhang et al. 2016b; Zeng et al. 2017). However, different medium pH values influence the FA profiles at different levels (Hall and Ratledge 1977). Similar scenarios were first observed in *R. toruloides* IIP-30 (formerly known as *R. glutinis* IIP-30) in the pH range of 3–6 (Johnson et al. 1992). Dias et al. (2016) also reported that medium pH values slightly altered FA profiles in *R. toruloides*. A slight MUFA percentage (OA) increased in the pH range 4.0–5.5. The PUFA percentages (LA and ALA) decreased at pH 4.5–5.5. Although the alteration in FA profiles did not show strong pH dependency, a specific pH range could assist in specific FA-rich lipid production based on their studies.

### Light

*Rhodotorula toruloides* is a promising strain for carotenoid production. The carotenoid and lipid biosynthesis pathways are related because acetyl-CoA molecules are shared as precursors (Bruder et al. 2019). As a critical photo-inducer, light can promote carotenoid production by increasing microbial growth and activity of enzymes essential for carotenoid biosynthesis (Frengova and Beshkova 2009). In addition, photo-induced reactive oxygen species (ROS) may be associated with the activation of carotenoid synthesis pathways through the transcription factor bZIP in *R. toruloides* (Lin et al. 2017). Pham et al. (2020) demonstrated that *R. toruloides* cells grown under light conditions showed higher POA (two-fold) and ALA (four-fold) contents of TFA compared to those grown under dark conditions. Their results indicate that light could be a helpful factor for controlling FA profiles in *R. toruloides*.

### Electro-fermentation

Electro-fermentation (EF) is a promising technique for improving the performance of bioprocesses that has been applied to increase lipid production in *R. toruloides* (Arbter et al. 2019). EF with the redox mediator Neutral Red (NR) caused a significant shift in the FA profile from OA to PA, resulting in a highly saturated FA composition (from 37 to 50%) in *R. toruloides* (Arbter et al. 2019). EF with NR is an excellent technique for producing highly saturated lipids as an alternative source of plant lipids such as coconut oil. In addition, EF with different media and strains may be applicable to *R. toruloides*.

## Conclusion

Numerous review articles have summarized the different strategies for maximizing lipid production in *R. toruloides*. However, few have summarized and compared the impact of each method on FA composition. Thus, this mini-review describes strategies and advanced progress for controlling the FA composition in *R. toruloudes* through metabolic engineering. In addition, this mini-review also summarized the parameters of culture conditions such as substrate utilization and bioprocess control that have impacts on FA profile in *R. toruloides*. *R. toruloides* exhibits flexibility in producing lipids that are rich in saturated FA, MUFA, PUFA, and even unusual FAs. Therefore, *R. toruloides* is a promising platform for producing specific FA lipids. Further studies are necessary to reveal the mechanisms underlying the described strategies and findings to precisely control FA synthesis and bioprocess in *R. toruloides* for specific FA lipid production.

## Data Availability

The data supporting the findings of this mini-review are available in the references cited within this article.

## Abbreviations

AA: Arachidic acid
ACP: Acyl carrier protein
ALA: α-Linolenic acid
ALD: Aldehyde dehydrogenase
ALE: Adaptive laboratory evolution
ARTP: Atmospheric and room temperature plasma
AT: Acyltransferase
ATMT: Agrobacterium tumefaciens-mediated transformation
C: Carbon
CG: Crude glycerol
DA: Docosanoic acid
DH: Dehydratase
DHA: Docosahexaenoic acid
EA: Erucic acid
EF: Electro-fermentation
ELO: Elongase
EO: Essential oil
ER: Enoyl reductase
ETA: Eicosatrienoic acid
FA: Fatty acid
FAD: Fatty acid desaturase
FAME: Fatty acid methyl ester
FAS: Fatty acid synthase
GLA: γ-Linolenic acid
KCS: Ketoacyl-CoA synthase
KR: Ketoacyl reductase
KS: Ketoacyl synthase
LA: Linoleic acid
MA: Myristic acid
MPT: Malonyl/palmitoyl transferase
MUFA: Monounsaturated fatty acid
N: Nitrogen
NA: Nervonic acid
NR: Neutral red
NTG: Nitrosoguanidine
OA: Oleic acid
ODFA: Odd-chain fatty acid
P: Phosphate
PA: Palmitic acid
POA: Palmitoleic acid
PT: Phosphopantetheine transferase
PUFA: Polyunsaturated fatty acid
ROS: Reactive oxygen species
Rt: Rhodotorula toruloides
S: Sulfate
SA: Stearic acid
TA: Tetracosanoic acid
TFA: Total fatty acid
UV: Ultraviolet
VFA: Volatile fatty acid
VLCFA: Very-long-chain fatty acid
